# Emergence of Leadership within a Homogeneous Group

**DOI:** 10.1371/journal.pone.0134222

**Published:** 2015-07-30

**Authors:** Brent E. Eskridge, Elizabeth Valle, Ingo Schlupp

**Affiliations:** 1 Department of Computer Science and Network Engineering, Southern Nazarene University, Bethany, Oklahoma, United States of America; 2 Department of Biology, Southern Nazarene University, Bethany, Oklahoma, United States of America; 3 Department of Biology, University of Oklahoma, Norman, Oklahoma, United States of America; Institut Pluridisciplinaire Hubert Curien, FRANCE

## Abstract

Large scale coordination without dominant, consistent leadership is frequent in nature. How individuals emerge from within the group as leaders, however transitory this position may be, has become an increasingly common question asked. This question is further complicated by the fact that in many of these aggregations, differences between individuals are minor and the group is largely considered to be homogeneous. In the simulations presented here, we investigate the emergence of leadership in the extreme situation in which all individuals are initially identical. Using a mathematical model developed using observations of natural systems, we show that the addition of a simple concept of leadership tendencies which is inspired by observations of natural systems and is affected by experience can produce distinct leaders and followers using a nonlinear feedback loop. Most importantly, our results show that small differences in experience can promote the rapid emergence of stable roles for leaders and followers. Our findings have implications for our understanding of adaptive behaviors in initially homogeneous groups, the role experience can play in shaping leadership tendencies, and the use of self-assessment in adapting behavior and, ultimately, self-role-assignment.

## Introduction

Many animals form large aggregations that have no apparent consistent leader, yet are capable of highly coordinated movements [1]. How this is possible has fascinated biologists for a long time. Which individuals of a group emerge as leaders, and why? Often all individuals of a large group are considered to be equal, but realistically, all individuals in a homogeneous group are not equal and even small differences may affect emerging leadership. Clearly individuals will differ based on traits like sex, age, and size. However, identifying these differences between individuals in a large group, and the impacts they have, is impractical. Nevertheless, some traits can be particularly relevant for the development of leaders. Of particular interest in this context are individual differences in experience. Other work has shown that differences in experience can shape an individual’s boldness, a trait often associated with leadership [2, 3], even when individuals are initially identical [4]. If an individual’s tendency to lead, or be successful in leading, is indeed shaped by experience, how do individuals respond and adapt to successes and failures in attempted leadership? Do individuals with different tendencies to lead respond to new experiences differently? Furthermore, what effects do the differences in the composition and size of the group have? These questions are relevant to many social animals, including humans, but are also pertinent in artificial systems, where individual units may acquire information that change their behavior [5–8].

The question of emergent leadership in biological systems has received much attention in recent years, including direct experimental [9, 10], broad theoretical [11], and agent-based modeling approaches [12, 13]. Previous work has frequently treated differences in behavioral dynamics, such as those often associated with leadership, as fixed (genetic) traits, but recent work has emphasized the potential role of experience in shaping these differences [4]. Most recently, Nakayama et al. [14] showed experimentally that leaders are less likely to respond to social experience in sticklebacks. Johnstone and Manica [15] previously provided the theoretical basis for an investigation of the effects of different intrinsic leadership probabilities on leader effectiveness using an *n*-player extension of the Battle of the Sexes game. However, their work uses an evolutionary approach in which intrinsic leadership probabilities, or strategies as they are called, are removed or added to the population based on their success as leaders. In contrast, the present study investigates the effects of success and failure on plastic, or adaptive, intrinsic leadership tendencies (denoted *LTs*). This adaptation of LTs in response to success and failure is likely to have important consequences for the behavior of groups and decision making processes within the groups. Despite the many simplifying assumptions made in the model, the study of adaptive LTs described here may also have consequences for our understanding of these processes in biological systems.

In the present study, we model the effects of LTs and individual experience on leadership using an agent-based collective decision-making model. Such models are becoming increasingly useful in understanding the behavior of complex social systems [16]. With this model, we add a simplified model of LTs to ask the following sets of questions: (1) How do individual LTs adapt in complex, anonymous interactions to experiences of success and failure? Do distinct and stable leaders and followers emerge? Can low LT individuals become successful leaders? (2) How much experience is required for individuals to differentiate into leaders and followers? (3) Once differentiation has occurred, how stable are the LTs? How often do they reverse? (4) How many high LT leaders can a group support?

## Materials and Methods

The collective decision-making model chosen for this simulation study was developed through observations of collective movement attempts in a group of ten white-faced capuchin monkeys [13, 17], and was later confirmed in observations of sheep groups ranging in size from 2–8 members [18]. It uses anonymous mimetism [19] and consists of three probabilistic interaction rules to govern the decision-making process involved in starting collective movements. The first rule governs when individuals in the group initiate a collective movement attempt. Once an initiation has occurred, the second rule governs when individuals in the group decide to follow the initiator. Lastly, the third rule governs when the initiating individual cancels the movement attempt. The equations corresponding to each rule are used to produce a time for each individual at which the associated event occurs. The individual with the earliest decision takes the appropriate action and the times for the remaining individuals are recalculated, taking into account the recent event. Note that this model only applies to the decision-making before an actual collective movement is made. As such, the work presented here does not try to model any spatial movement in the environment.

The first rule assumes that all individuals within the group can initiate a collective movement attempt. While this assumption may not hold for groups with dominant leaders, studies have shown that it is a viable assumption for animal groups with distributed leadership, such as the capuchin monkeys used in the model’s development, and is frequently used in biologically-inspired robot swarms [20]. All individuals initiate according to the base rate of 1/*τ*_*o*_ where the time constant *τ*_*o*_ was determined through observation (see Table 1). Once an individual initiates a movement attempt, no other individuals may attempt to initiate a movement. Future work includes removing this restriction.

**Table 1:** Model parameters determined through direct observation of collective movement attempts in white-faced capuchin monkeys.

Parameter	Value
*τ*_*o*_	1290
*α*_*c*_	0.009
*γ*_*c*_	2.0
*ɛ*_*c*_	2.3
*α*_*f*_	162.3
*β*_*f*_	75.4

The second rule describes the rate at which followers join the collective movement attempt. Since the model assumes global communication, once an individual initiates a collective movement, all the other individuals in the group are assumed to have observed the initiation attempt and have the opportunity to follow the initiator. As the number of individuals following the initiator increases, the rate at which individuals join the movement attempt also increases. Individuals follow at the rate 1/*τ*_*r*_, where the time variable *τ*_*r*_ is calculated by the following equation:

$$\tau_{r}=\alpha_{f}+\beta_{f}\frac{\left(N-r\right)}{r}$$ (1)

where *α*_*f*_ and *β*_*f*_ are constants determined through direct observation of capuchin monkey following events (see Table 1), *N* is the total number of individuals in the group, and *r* is the number of individuals following the initiator.

The third rule describes the fact that not all initiation attempts are successful, as initiators often cancel their initiation attempt and return to the group. As the number of individuals following the initiator increases, the rate at which the initiator cancels an initiation decreases. This cancellation rate is calculated using the following equation:

$$C_{r}=\frac{\alpha_{c}}{1+{\left(r/\gamma_{c}\right)}^{\varepsilon_{c}}}$$ (2)

where *α*_*c*_, *γ*_*c*_, and *ɛ*_*c*_ are constants determined through direct observation of capuchin monkey canceling events (see Table 1), and *r* is the number of individuals following the initiator. Note that once an individual decides to follow an initiator, they may not alter their decision and will follow until all individuals are following the initiator or the initiator cancels. Simulations of the model include the implicit assumption that a successful collective movement requires all of the members of the group to participate, since there is a non-zero probability of canceling even if all but one member participates. While this is not necessarily the case in all natural systems, the decision-making processes for cohesive, collective movements are the primary objective of this work and, as such, incomplete movements are considered failures.

### Adaptive Leadership Tendencies

To investigate the effects of altering the rate at which individuals initiate a movement, follow an initiator, and cancel a movement, Gautrais added an individual-specific constant, referred to as a “*k* factor,” to the rate calculations of the collective decision-making model [13]. This *k* factor does not refer to a specific biological attribute. It was merely a mechanism to introduce over-initiating and over/under-following. Initiation attempts were now calculated at the rate of *k*/*τ*_*o*_, and the following and canceling rate calculations were modified as follows:

$$\tau_{r}=\frac{1}{k}\left(\alpha_{f}+\beta_{f}\frac{N-r}{r}\right)$$ (3)

$$C_{r}=k\left(\frac{\alpha_{c}}{1+{\left(r/\gamma_{c}\right)}^{\varepsilon_{c}}}\right)$$ (4)

where the variables are defined as before. In the initial study, the value of the constant was arbitrarily chosen for evaluation models and had no biological motivation. Since this *k* factor can either increase or decrease the three decision-making rates, it was an ideal means with which the effects of an adaptive LT could be incorporated into the model.

Three important points were considered in integrating LT with the collective movement model. First, intrinsic leadership tendencies have been observed in natural systems to affect the events used in this model in different ways. For example, a high LT individual should have a higher initiation rate and lower following and canceling rates since consistent leaders are frequently less sensitive to the actions of others in the group [2, 21]. On the other hand, a low LT individual should have a lower initiation rate and higher following and canceling rates since followers are frequently more sensitive to the actions of others. Second, the magnitude with which a low LT value affects the model should be the same as a high LT value so as not to bias the model. Since *k* had a non-inclusive lower limit of zero, the non-inclusive upper limit of two was chosen to ensure balance (*k* ∈ [0, 2]). In the simulations described below, LT values were limited to the range [0.1,0.9] to ensure these limits were satisfied. Lastly, although neither the original model, nor the observations on which the model was based, discuss individual tendencies towards leadership of the individual animals involved, we assumed that the observed group members had LTs represented by an internal variable *L*_*i*_, ranging from low (*L*_*i*_ = 0.1) to high (*L*_*i*_ = 0.9). To convert this individual LT into behavioral rates, the “*k* factor” was calculated using the following nonlinear sigmoid function:

$$k=2{\left(1+{\text{e}}^{\left(0.5-L^{\prime }\right)\times 10}\right)}^{-1}$$ (5)

where *L*′ is *L* for initiating decisions and is 1 − *L* for canceling and following decisions. A default moderate LT (*L* = 0.5) recovers the original model. The choice of this sigmoid function was made after exploratory evaluations using a variety of different functions and does not have any explicit biological basis, although studies have shown that nonlinear feedback loops contribute to differentiation of individuals in groups [22].

**Fig 1:**
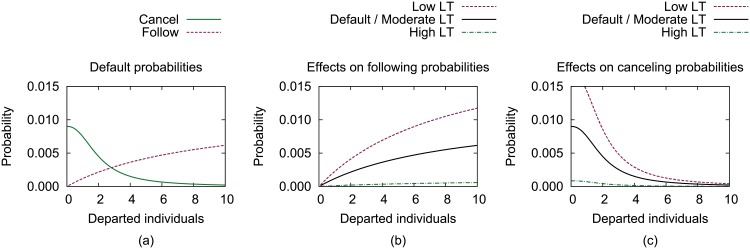
The effects of LT on the following and canceling rates are shown. The *(a)* default rates from the original model are shown on the left, while the *(b)* following and *(c)* canceling rates are shown in the middle and right, respectively. The effects of LT values on the following and canceling rates are shown for initially low, moderate, and high LT values. The effect of the LT value on the initiation rate is not shown since initiations are calculated using a constant value and are not dependent on the number of departed individuals.

Fig 1 depicts the effects of LT on the following and canceling rates as a function of the number individuals that have departed as members of the collective movement. The default rates from the original model are shown on the left, while the following and canceling rates are shown in the middle and right, respectively. In the original model, the likelihood of an initiator canceling the movement attempt is initially relatively high and is more likely to happen than individuals deciding to follow the initiator. However, once a threshold number of individuals follow the initiator, two followers plus the initiator in this case, the likelihood of the initiator canceling is less likely than subsequent following events from other individuals.

As in observations of natural systems, high LT individuals in our simulations are less likely to follow and cancel, whereas low LT individuals are more likely to follow and cancel. Also, high LT and low LT values affect the rates with equal magnitude, preventing an inherent bias in the model. Lastly, moderate LT values (*L* = 0.5) resulted in no change to the decision rates. The effects of LT values on the initiation rate are not shown graphically as they are not dependent on the number of departed individuals. Although valid LT values could range from [0, 1], LT values were limited to the range [0.1,0.9] in these simulations to avoid divide-by-zero situations for *k*.

The initiating individual’s LT value was updated after every initiation attempt using the following linear update (or learning) rule used in work on both biological and artificial systems [23–25]:

$$L_{i,t+1}=L_{i,t}\left(1-\lambda \right)+\lambda r$$ (6)

where *L*_*i*,*t*_ was the initiator *i*’s LT for the current movement, *L*_*i*,*t*+1_ was the LT for the next movement, *λ* was the rate at which updates changed the LT value with a range of *λ* ∈ [0, 1], and *r* was the reinforcement value used to update the LT value. If an update to the LT value exceeded the specified range, the excess was truncated to ensure the value remained within the range. The rule did not bias LT values towards one value or another since the magnitudes of the effect of success and failure on the LT value were the same. When *λ* was low, the LT was primarily determined through long-term historical success and changes were minor. When *λ* was high, the LT was primarily determined through short-term success, namely the last initiation attempt, and changes from one attempt to the next were significant. For the simulations described in this work, a low value of *λ* was chosen (*λ* = 0.02) to emphasize long-term initiation success. For successful initiations, the reinforcement value was *r* = 1, while it was *r* = 0 for unsuccessful initiations.

### Numerical Implementation

Numerical simulations of the collective decision-making model were implemented in Java using the same general algorithm as in previous work [13] (see Algorithm 1 in S1 Table). The time of each event was calculated as a random number drawn from an exponential distribution using the appropriate rate as calculated by the model’s rate equations (see Eqs 3 and 4). As such, the simulations use continuous time events, and not discrete time events or probabilities of decisions occurring during a discrete time step. The original model was only evaluated with a group size of 10, but other results have shown that the success of collective movement initiations increases as the group size is increased, with diminishing effects beyond a group size of 100 [26]. Therefore, evaluating different group sizes presented an opportunity to evaluate the effects of LT with different group dynamics. To evaluate the effect of the initial LT value, treatments were performed using the following LT values for all individuals within a group: low (*L*_*i*_ = 0.2), moderate (*L*_*i*_ = 0.5), and high (*L*_*i*_ = 0.8). Within each treatment, group sizes ranging from 10 to 150 individuals were used. Fifty evaluations were performed for each group size, each with a different random seed with evaluations between treatments having the same initial conditions, including the random seed. A single evaluation consisted of 2,000 × *N* movement attempts, where *N* was the group size. This value was chosen to ensure there were sufficient initiation attempts (i.e., simulations) for LT values to differentiate. Each simulation constituted a single initiation attempt at a collective movement and ended in either success (all individuals participating in the movement) or the initiator canceling. Individual LT values persisted from one simulation to the next and were reset at the beginning of each evaluation. The values used for the model parameters were the same as those used in the original model [13, 17]. All source code for the collective movement simulator, including scripts to perform the simulations and analyze the results, is freely available as a public GitHub repository: https://github.com/snucsne/bio-inspired-leadership.

To analyze trends in LT values of successive attempts in an evaluation, the R package strucchange was used [27]. This software package identifies structural shifts in time series data. In our simulations, these shifts, called *breakpoints*, represent a LT value transition. Since LT values were not constant and the analysis produces a linear approximation of a portion of LT value time series, a LT was defined to be high if a segment of the LT value had a mean value greater than or equal to 0.775. This threshold was high enough such that evaluations using an initially high LT value did not affect any analysis.

## Results

To answer the research questions previously identified (see Introduction), the raw results from the evaluations were analyzed in a number of ways. First, the evaluation results were analyzed to answer the question regarding the emergence of distinct leaders and followers. The analysis indicates that not only do distinct leader and follower LT values emerge, but their emergence results in statistically significantly higher initiation success (see Emergence of Leaders and Followers). Second, the evaluation results were analyzed to determine the amount of experience required for the LT values to differentiate. This analysis indicates that relatively few experiences were required for differentiation (see Experience Required for Differentiation). Next, an analysis was performed to determine the stability of the distinct LT values after differentiation (see Stability of LT Values). Lastly, relative percentage of high LT leaders supported by a group was analyzed (see Distribution of LT Values).

### Emergence of Leaders and Followers

**Fig 2:**
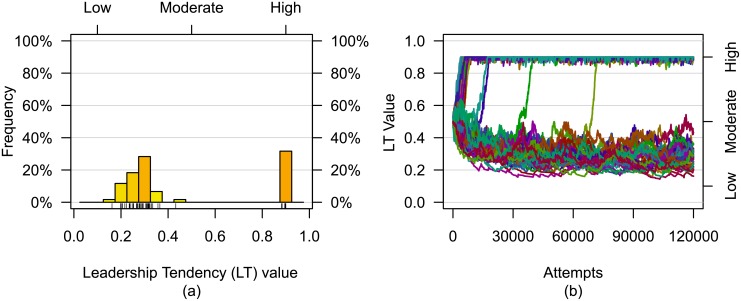
A *(a)* representative final LT value distribution and *(b)* history of LT value changes resulting from experience are shown. This evaluation used a group of size *N* = 60 with an initially moderate LT value.

In all evaluations for all group sizes, individual LT values differentiated into distinct high LT leader values and low LT follower values although all LT values were initially the same. Fig 2 presents a representative illustration of the final LT value distribution and the associated history of LT value changes due to experience gained within a single evaluation. Note that stable moderate LT values did not emerge in this evaluation. In fact, stable moderate LT values did not emerge in any of the evaluations for the set of parameters used.

**Fig 3:**
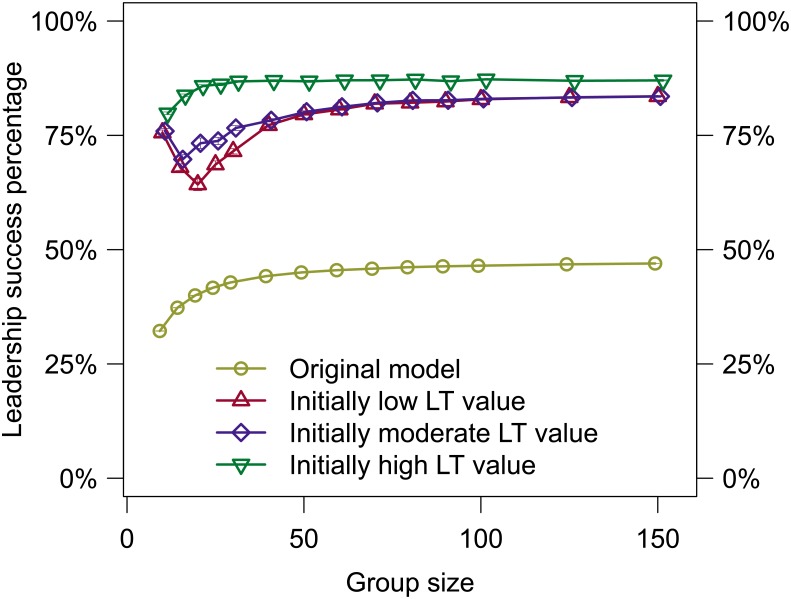
The mean leadership success in initiating collective movements is shown for all treatments. All individuals within the group were given the same initial LT value, but individuals adapted their LT value based on experience in initiating collective movements attempts (mean/SE, but is small and may not be visible).

Fig 3 shows the success of initiators in leading collective movements (i.e., movements that ended with all individuals participating) over the entire evaluation, not just after differentiation. All simulations using LT, regardless of the initial value, performed statistically significantly better than simulations using the original model (Student’s t-test, *p* < < 0.001, see S2 Table for a complete statistical analysis). As with the original model, leadership success increased as the group size increased [26]. However, simulations using an initially low and moderate LT value predicted an initial loss in success when increasing the group size from 10 to 15.

### Experience Required for Differentiation

**Fig 4:**
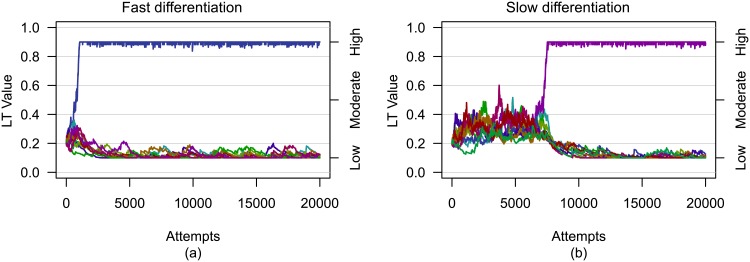
Histories of LT value differentiation for *(a)* fast and *(b)* slow evaluations. The evaluations both used a group size of 10 and initially low LT values. In both evaluations, LT values were distributed at the extremes of the allowed range of values after differentiation, but the number of simulated movement attempts required for differentiation differed between evaluations.

**Fig 5:**
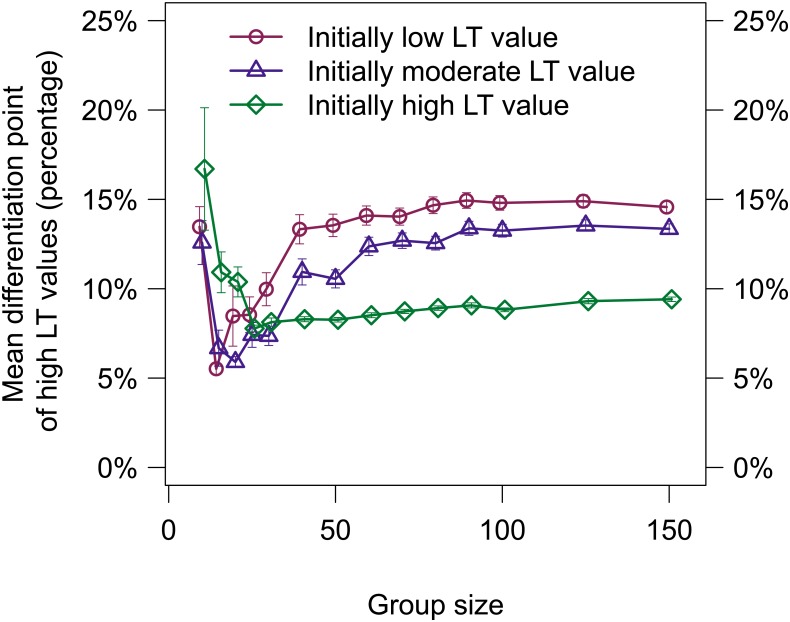
The mean percentage of initiation attempts required for LT value differentiation are shown (mean/SE). Differentiation is defined as the percentage of total possible initiation attempts in an evaluation required for an individual *i*’s LT value to emerge as high.

Fig 4 depicts the LT value histories for two different evaluations using initially low LT values and a group size of 10. In the first evaluation, an individual differentiated into a high LT individual rapidly (under 1,000 simulated attempts), while a similar agent took over 7,000 attempts to differentiate in the second. Fig 5 shows the mean number of initiation attempts as a percentage of the total number of simulations for individual LT values to differentiate into distinct high and low values. As noted above, a LT value was defined as having emerged as high when the individual *i*’s LT value transitioned such that *L*_*i*_ ≥ 0.775 as determined by a breakpoint analysis using the R package strucchange [27]. The mean number of initiations required for differentiation of LT values was less than 15% of the total number of initiation attempts for evaluations of all group sizes and initial LT values except for evaluations with a group size of 10 and initially high LT values, which required 17±3% (see S3 Table for a complete statistical analysis). For a group size of 10, evaluations using initially high LT values took longer to differentiate than evaluations using initially low or moderate LT values. However, for group sizes of 40 and larger, evaluations using initially high LT values differentiated faster and the overall number of initiation attempts required for differentiation as a percentage of the total number of initiation attempts remained consistent.

### Stability of LT Values

**Fig 6:**
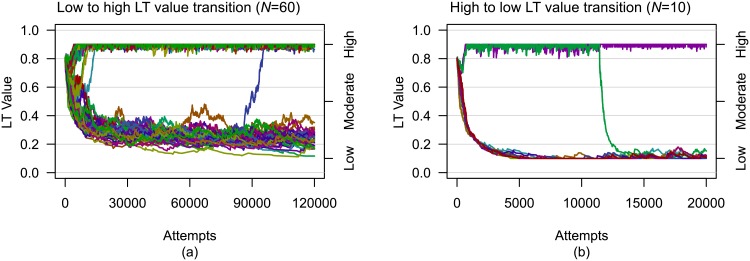
Two representative LT value histories depicting transitions between extremes are shown. Although LT values were relatively stable after the entire group had adapted, transitions between the extremes did occur. The more common transition was *(a)* from low to high LT value (*N* = 60 and initially high LT values). However, a few rare *(b)* high to low LT value transitions were observed (*N* = 10 and initially high LT values).

**Fig 7:**
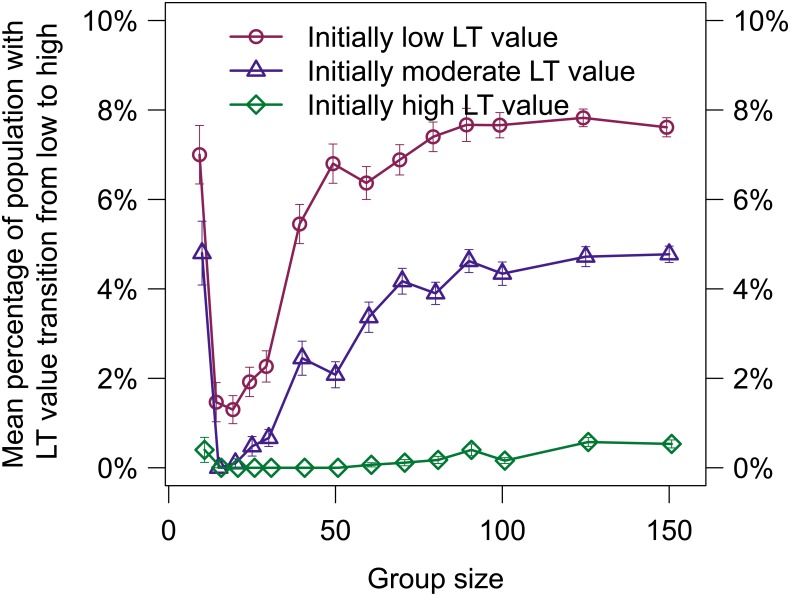
The mean percentage of LT values that transitioned from low to high are shown (mean/SE). Transitions were defined as LT value changes occurring after the initial differentiation.

Once individual LT values differentiated, the majority remained stable with only minor fluctuations. There were a number of instances of low LT values transitioning to high LT values well after the initial differentiation of personalities, but only two instances of a high LT value transitioning to low (see Fig 6). Fig 7 shows the mean percentage of the evaluation group size in which a LT value transitioned from low to high. Excluding a group size of 15, evaluations with an initially high LT value were more stable than evaluations with initially low and moderate LT values (see S4 Table for a complete statistical analysis).

### Distribution of LT Values

**Fig 8:**
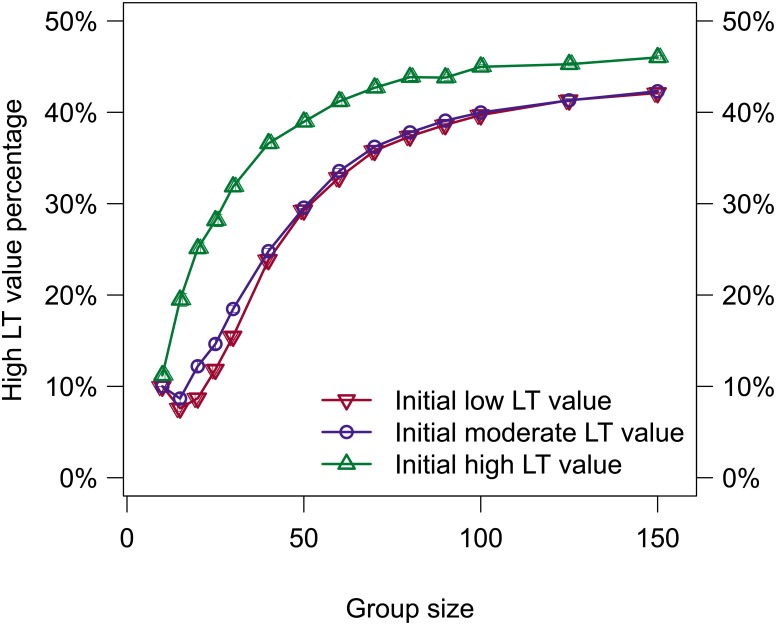
The mean percentage of high LT value individuals at the end of an evaluation are shown (mean/SE).

Fig 8 shows the mean percentage of high LT values at the end of an evaluation for all initial LT values. For group sizes beyond 10 individuals, evaluations using an initially high LT value supported statistically significantly more high LT individuals than evaluations using the other initial LT values. Furthermore, larger group sizes were able to support a greater percentage of high LT individuals, excluding a group size of 15 for initially low and moderate LT values.

## Discussion

Studying the emergence of leadership in groups of seemingly equal individuals is an important topic both in natural and artificial systems. An improved understanding of leadership in such situations would enable researchers to better predict individual and group behaviors in natural systems and better design artificial systems to exhibit preferred behaviors. In our simulations, all individuals started with identical initial leadership tendency (LT) values, but small differences in experience had profound effects on the emergence and maintenance of leadership. Positive experiences made it more likely that individuals become leaders. Refuted attempts at leadership caused the opposite: a failed leader was very likely to become—and remain—a follower. While such experience-based adaptations occur frequently in nature and the design of the system promoted such situations with the reward structure, the stability of individual LT values, and of the system as a whole, was particularly noteworthy. Exploratory simulations performed in the development of this model and since show that this stability is rare and that the nonlinearity of Eq 5 was essential to the differentiation and stability of the LT values.

Given the static environment, once high LT value individuals emerged, they began to dominate initiation attempts, as would be expected given the model. However, the differentiation of high and low LT value individuals happened rapidly, especially given that LT value updates emphasized repeated, long-term experiences, and not short-term successes or failures. Furthermore, after the initial differentiation, individual LT values were stable and rarely switched from high to low and vice-versa. The simulations predict that group size is an important factor, with larger groups allowing for a greater percentage of high LT value individuals to persist. In this agent-based model, early experience was very predictive of future behavior. This is consistent with observations of many social animals in which individual experience is known to play a role in leadership. An example is the role that experienced worker bees play in finding new hives: apparently self-assigned, older foragers respond to the perceived need to form a new swarm and scout for suitable tree holes. Parallel to what our simulations predict, they then compete with other scouts for leadership and try to recruit other bees to follow them [28, 29]. In another example, differences in experiences in risk-taking behavior have also been shown to result in a dimorphism of personality types, often associated with leadership, in simulations using a hawk-dove game and similar feedback mechanisms [30]. Several other studies use related concepts to address role assignment as it relates to hierarchy formation [31–33].

Unlike observations of natural systems, this agent-based model did not produce stable moderate LT values. Our analysis leads us to conclude that this is due to the nonlinear sigmoid function used to compute the “*k*” factor that modifies the rate equations (see Eq 5). The slope at the inflection point (*L* = 0.5) is such that small perturbations in the LT value at moderate levels have significant effects on the rate equations, while such perturbations at the extremes produce negligible effects. As such, the series of successful and failed experiences that would be required to produce a stable moderate LT value are highly unlikely, especially given that small variations in LT values in a group produce rapid differentiation into extreme LT values. However, moderate LT values may prove to be stable in other areas of the parameter space that these simulations did not evaluate.

Overall, distinct roles can improve the performance of the group as a whole with better outcomes for the individual (see Fig 3) [34–36], and these simulations predict that this role assignment can happen without a priori differences, but instead be based only on adaptations resulting from individual experience. At present, whether or not such situations exist in natural systems appears to be an open question as we have been unable to find evidence for or against it in the literature. While the challenge of a biological experiment to evaluate this prediction is significant, the benefits have the potential to be equally significant.

In our static simulation environment, individuals evaluate their own performance and self-assign themselves into distinct roles based on their success or failure, where they tend to remain. However, in a more dynamic environment such assignments would be more transitory [37]. In the context of a team of autonomous robots, consider an urban search and rescue operation. Given the unpredictable and dynamic nature of the environment, there may not be enough information for a human operator to effectively assign robots into different roles a priori. For example, while one robot could have a superior vision system, dust and debris could render the vision system useless, and enable a robot with a superior infrared vision system to be a more effective leader. These simulations predict that a system could be designed so that the robot team’s leadership emerges by way of direct experience in the environment. A similar situation could be envisioned for a natural system in which different individuals have strengths in different environmental situations and tasks.

As previously discussed, several studies of natural systems have shown that differentiation into roles can, among other benefits, improve a group’s success. The collective decision-making model used here produced similar predictions (see Fig 3). Evaluations in which individual LT values were allowed to differentiate into high and low LT values predicted far higher initiation success than those in which LT values were nonexistent or fixed, in some group sizes doubling the success rate. This improved success rate is due to both high and low LT values being present in the group. An individual with a higher LT value was more likely to initiate movements due to a higher initiation probability. Furthermore, since high LT value individuals were less responsive to other individuals within the group, they were less likely to cancel, similar to findings in natural systems [21]. In contrast, low LT value individuals were more likely to follow an initiator, providing the initiator with more followers faster and reducing the probability that the initiator would cancel.

To ensure that the addition of LT values in and of themselves did not bias the results, we performed evaluations with individuals having fixed LT values of low, moderate, and high (see S1 Fig). The results were identical to evaluations using the original model, which did not include LT values. If all individuals had high LT values, then the initiator was less likely to cancel, but all the potential followers were also less likely to follow. On the other hand, if all individuals had low LT values, potential followers were more likely to follow, but the initiator was also more likely to cancel. It was the combination of both high and low LT values that produced improved initiation success.

Since the differentiation of LT values was essential to the improved initiation success, reducing the amount of experience needed to differentiate directly impacts the initiation success. Although a larger *λ* value could be used to increase the rate at which individual LT values changed, we were more interested to learn if the choice of the initial LT value affected the amount of experience needed for differentiation. Fig 5 shows that for groups of 40 or less, the differentiation rates for different initial LT values were not consistent. However, for groups of 50 or more, the differentiation rates were consistent, with an ordering from fastest to slowest of high, moderate, and low initial LT values. Future work includes investigations into how best to reduce the time needed for differentiation.

In a number of cases, individuals having low LT values transitioned to high LT values (see the left plot in Fig 6 for an example). Fig 7 shows that evaluations with an initially high LT value that individual LT values would be far more stable than evaluations with an initial LT value of low or moderate, and rarely exhibited transitions from a low LT value to a high value. This finding is in agreement with other simulations that predict as an individual gains more experience, success has a greater effect than failure on their behavior [38].

Only two instances of an individual with a high LT value transitioning to a low value were observed, both of which occurred in evaluations in which initial LT values were high (see the right plot in Fig 6 for an example). This was most likely due to the inability of the population to support such a large number of initially high LT value individuals over the long term, but able to support a large number in the short term.

The fact that the initial LT value affected the mean percentage of high LT value individuals in the group was particularly surprising (see Fig 8). Evaluations with an initially high LT value consistently predicted greater percentages of high LT value individuals than initial LT values of low for groups larger than 15, whereas evaluations with initial LT values of low and moderate predict similar high LT value percentages for every group size. After an in depth analysis of the results, we hypothesize that the theoretical maximum percentage of high LT value individuals that are supportable by a group is not dependent on the initial LT value, despite what Fig 8 may indicate. Rather, we argue that evaluations containing individuals that initially had high LT values achieved a higher percentage of high LT value individuals due to the fact that it is easier for individuals with high LT values to transition to low LT values than the reverse. Since all individuals in the group started with high LT values, they only needed to experience enough success to maintain their LT value whereas other evaluations required individuals to experience long-term success to transition to a high LT value. There is precedence for individuals with little experience having a higher tendency for leadership [39].

Of particular interest is the fact that the trends shown in Fig 8 for the percentages of high LT value individuals match those shown in Fig 3 for the leadership success percentages, including the dip present for a group size of 15 for both low and moderate initial LT values. At present we do not know if there is an actual correlation between these two values and more investigation is warranted.

The model, as used here, has a number of limitations, which were primarily self-imposed to simplify the simulations. First and foremost is the assumption of global communication. For the large group sizes used here, this assumption is impractical, but simplified the model and the analysis. Future work includes removing this assumption and using only local communication. Second, the model classifies success as the initiator recruiting all possible followers and does not allow for partial success. Other models allow for partial success, but require more tuning [40]. Future work includes using a lower threshold for recruitment success and including alternative success criteria such as using an environmentally preferred direction of movement or destination location. Of particular interest is how leadership changes in response to environmental changes in success criteria [37].

## Conclusions

In summary, this work highlights the significant role experience can play in the emergence of distinct leadership tendencies (LTs) in a collective decision-making system, specifically those related to leaders and followers. In the simulations presented here, experience gained in successful or failed initiation attempts in a collective decision-making process produced changes in an individual’s tendency to lead or follow, resulting in a stable and rapid differentiation into distinct LT values. These LT values were then used to determine the likelihood of success in future initiation attempts when individuals assumed the roles of leaders or followers. Although these LT values were stable, they were not fixed and a number of individualss were observed transitioning from a low LT value, indicating a role of follower, to a high LT value, indicating a role of leader.

## Supporting Information

S1 Fig The leadership success in initiating collective decisions is shown for fixed LT values. All individuals within the group shared the same, fixed LT value. All seven LT value values produced the same results, showing that no single value produces a higher success percentage.
(PDF)

S2 Fig Alternative sigmoid functions used to calculate the “*k* factor”. (PDF)

S3 Fig Representative histories for LT value changes resulting from experience for alternative methods of calculating the “*k* factor” are shown. (a) A linear function. (b)-(f) A sigmoid function with different *a* coefficients. (d) Sigmoid function used in the principal paper.
(PDF)

S4 Fig The mean leadership success in initiating collective movements for alternative methods of calculating the “*k* factor” are shown. (a) A linear function. (b)-(f) A sigmoid function with different *a* coefficients. (d) Sigmoid function used in the principal paper.
(PDF)

S1 Table The collective decision-making simulation algorithm. The algorithm was developed in conjunction with the decision-making model for the original work and was rewritten for use in this work.
(PDF)

S2 Table A full statistical analysis of the mean success percentage of initiators. (PDF)

S3 Table A full statistical analysis of the mean percentage of initiation attempts required for differentiation. (PDF)

S4 Table A full statistical analysis of low to high LT value transitions. (PDF)

S1 Text Explanation of simulations using fixed LT values. Additional simulations were performed using fixed LT values to ensure that the modification made to add LT values to the collective decision-making model did not bias the model towards one particular LT value.
(PDF)

S2 Text Explanation of difficulty in achieving differentiation. This text presents supporting results regarding the challenge in developing a model in which LT value differentiation is stable in a constant environment.
(PDF)
