# Supplementary figures and images for: Emergence of Leadership within a Homogeneous Group
Source: PLoS One. 2015 Jul 30;10(7):e0134222. doi: 10.1371/journal.pone.0134222 (PMC4520564; doi:10.1371/journal.pone.0134222)

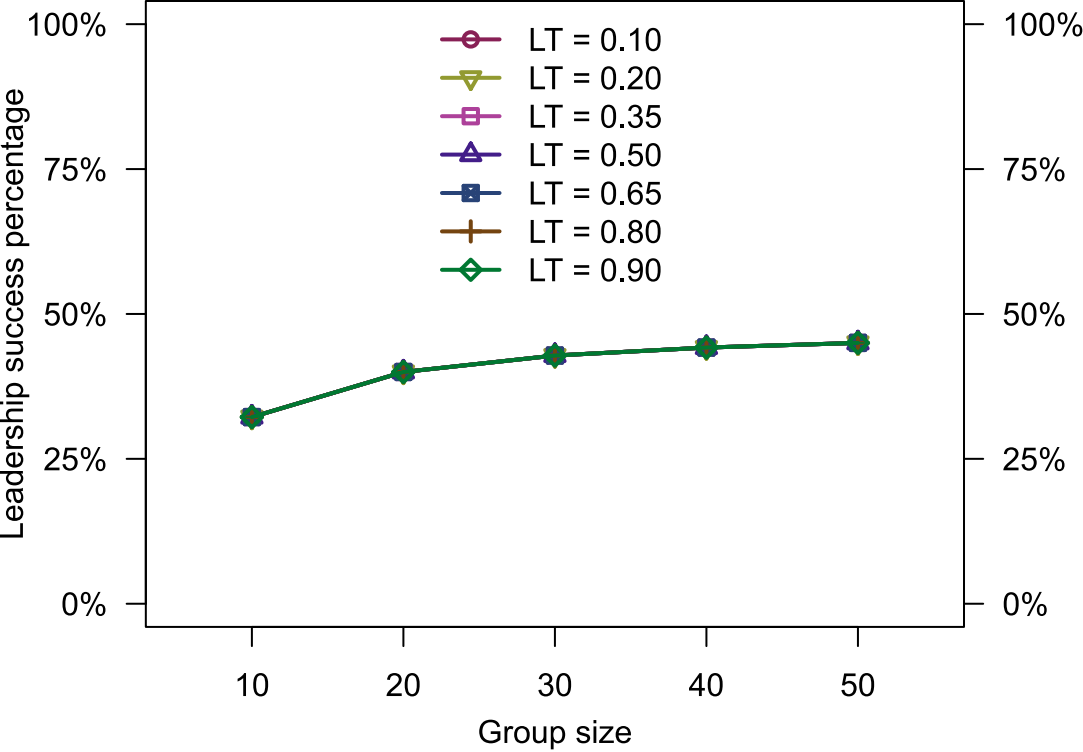

Supplement: S1 Fig — All individuals within the group shared the same, fixed LT value. All seven LT value values produced the same results, showing that no single value produces a higher success percentage. (PDF) [file pone.0134222.s001.pdf]

Sigmoid ( $a=5$ )  
Sigmoid ( $a=7$ )  
Sigmoid ( $a=10$ )

Sigmoid ( $a=15$ )  
Sigmoid ( $a=20$ )

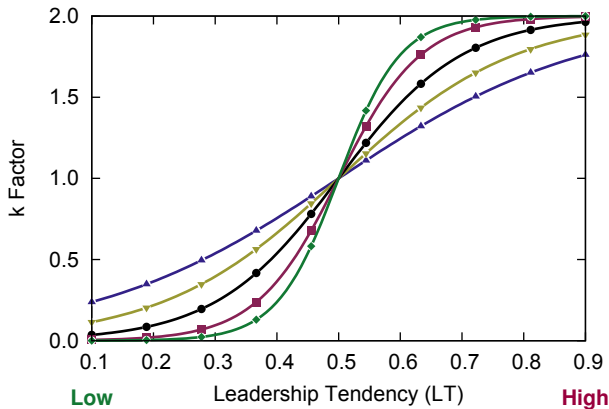

Supplement: S2 Fig — (PDF) [file pone.0134222.s002.pdf]

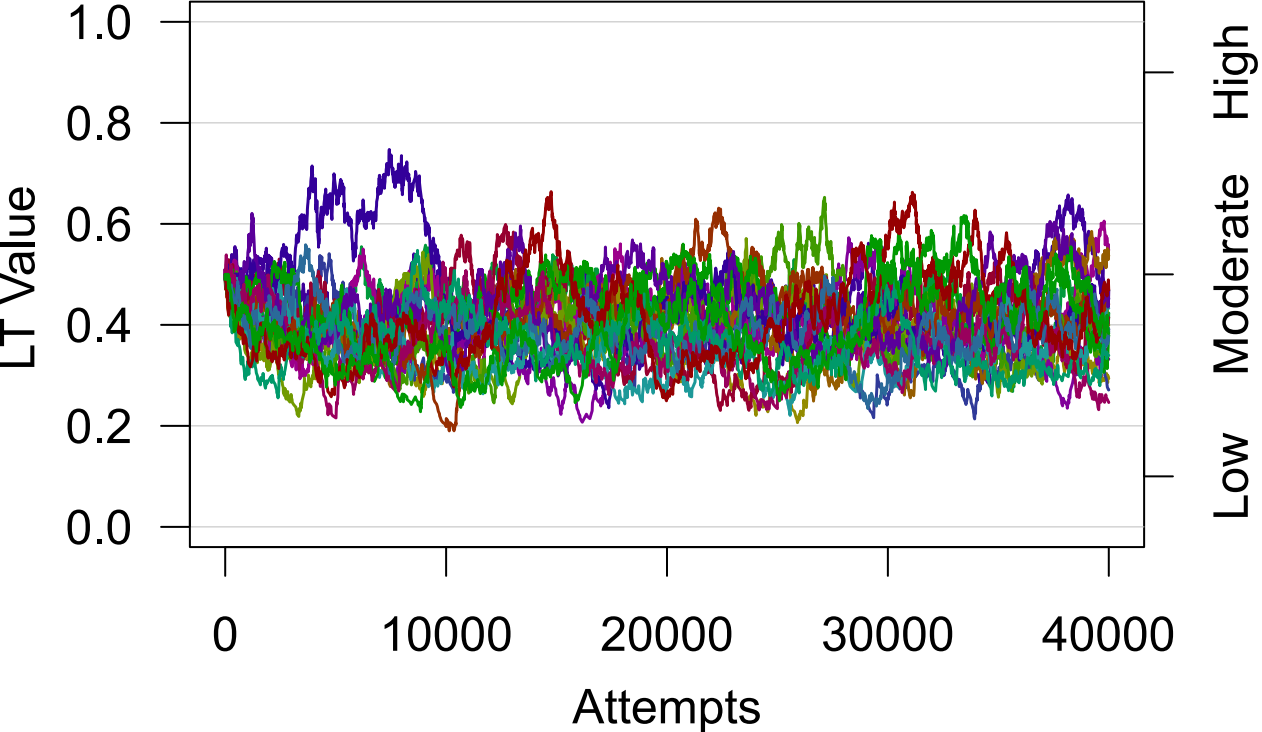

(a) Linear

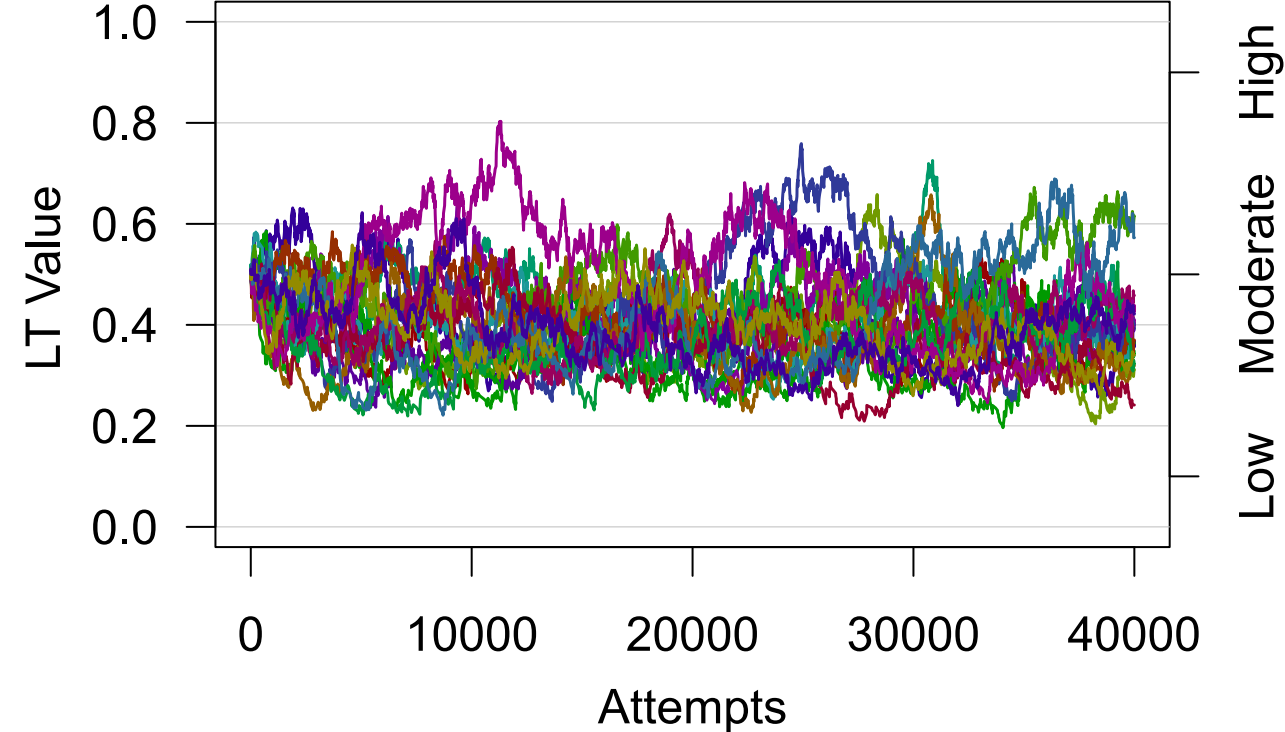

(b) Sigmoid ( $a = 5$ )

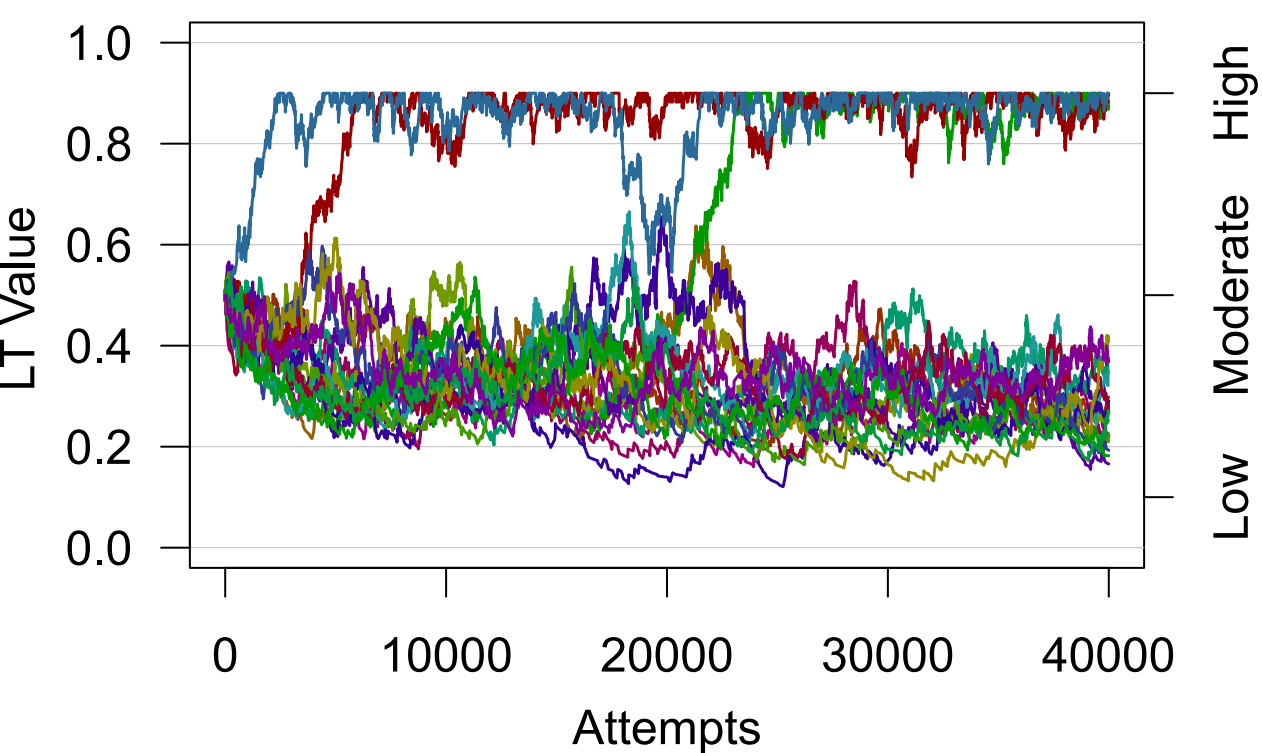

(c) Sigmoid ( $a = 7$ )

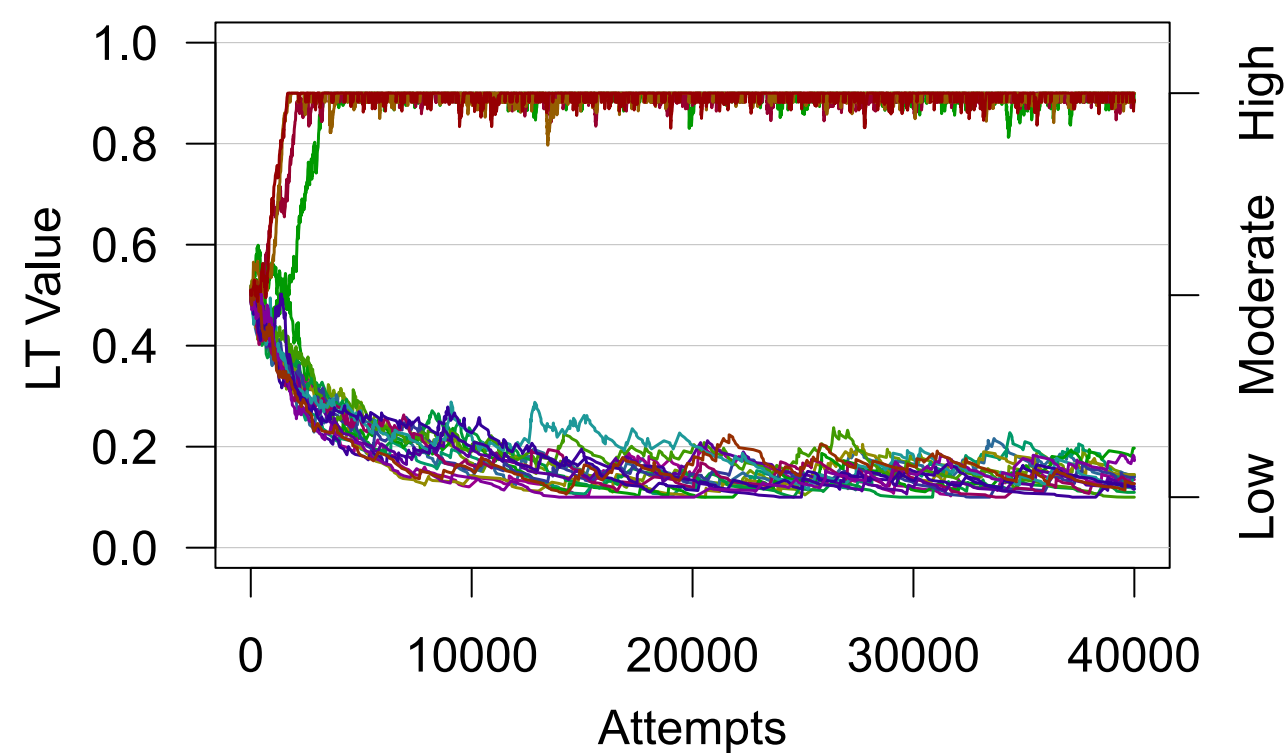

(d) Sigmoid ( $a = 10$ )

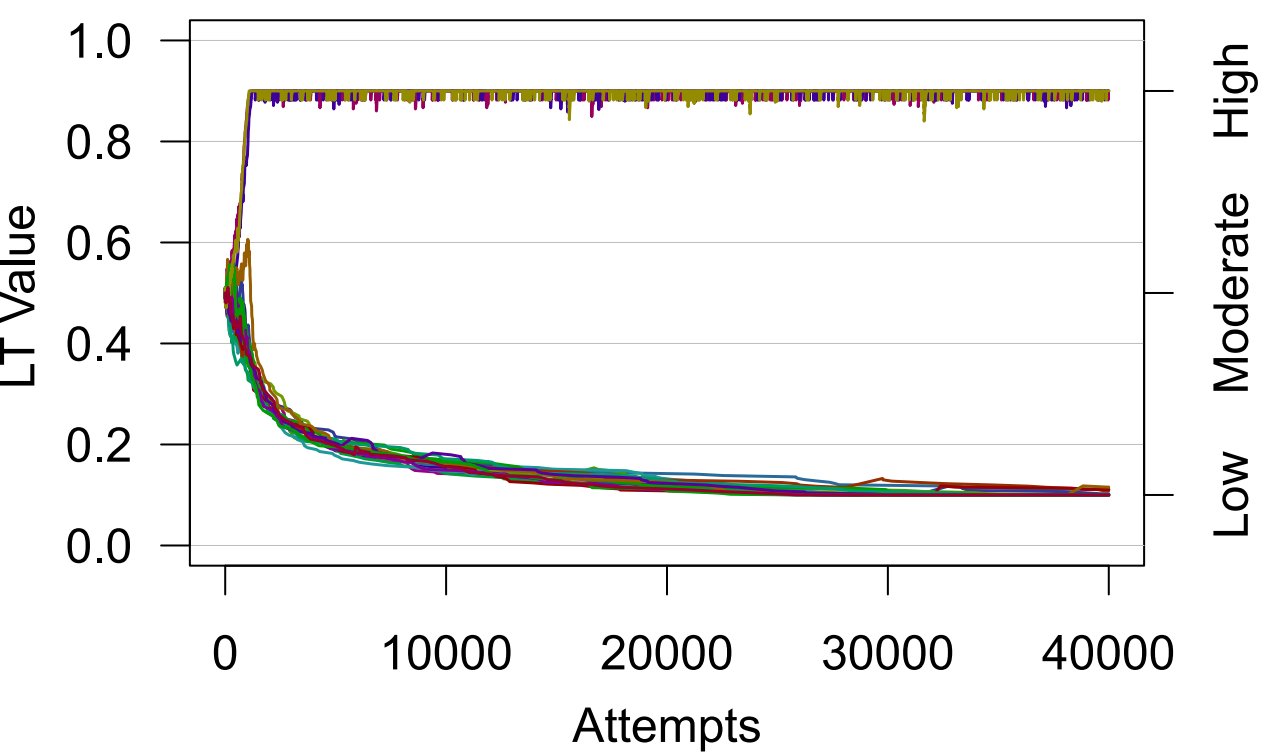

(e) Sigmoid ( $a = 15$ )

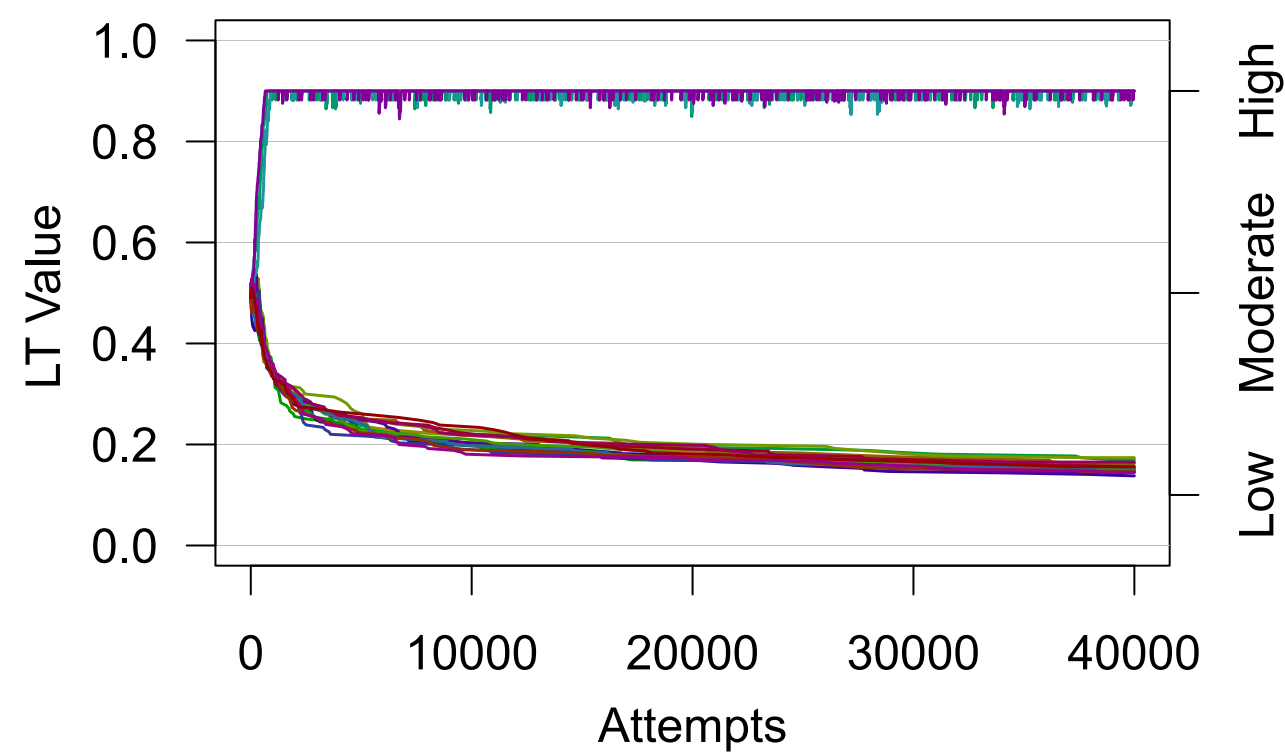

(f) Sigmoid ( $a = 20$ )

Supplement: S3 Fig — (a) A linear function. (b)-(f) A sigmoid function with different a coefficients. (d) Sigmoid function used in the principal paper. (PDF) [file pone.0134222.s003.pdf]

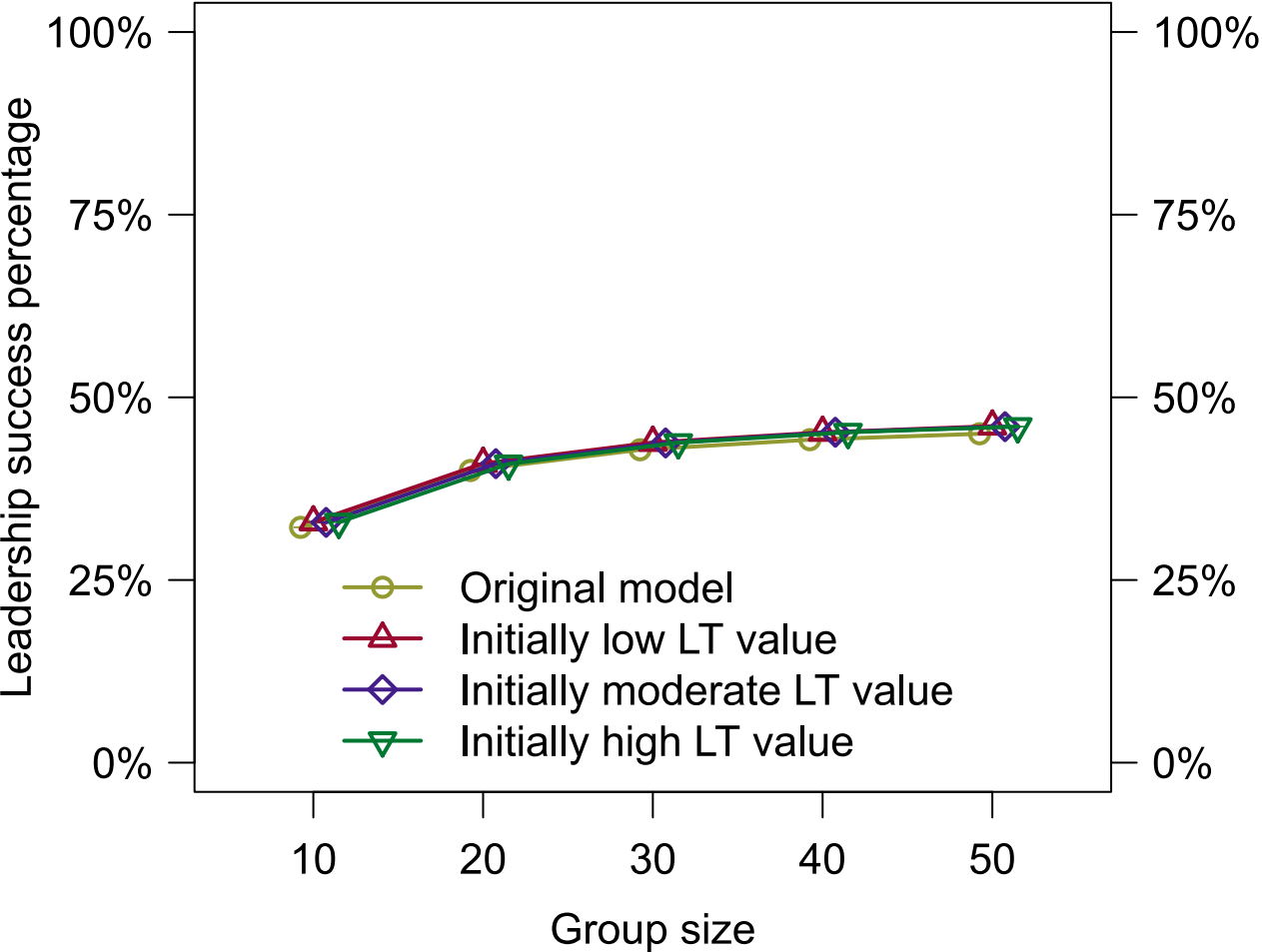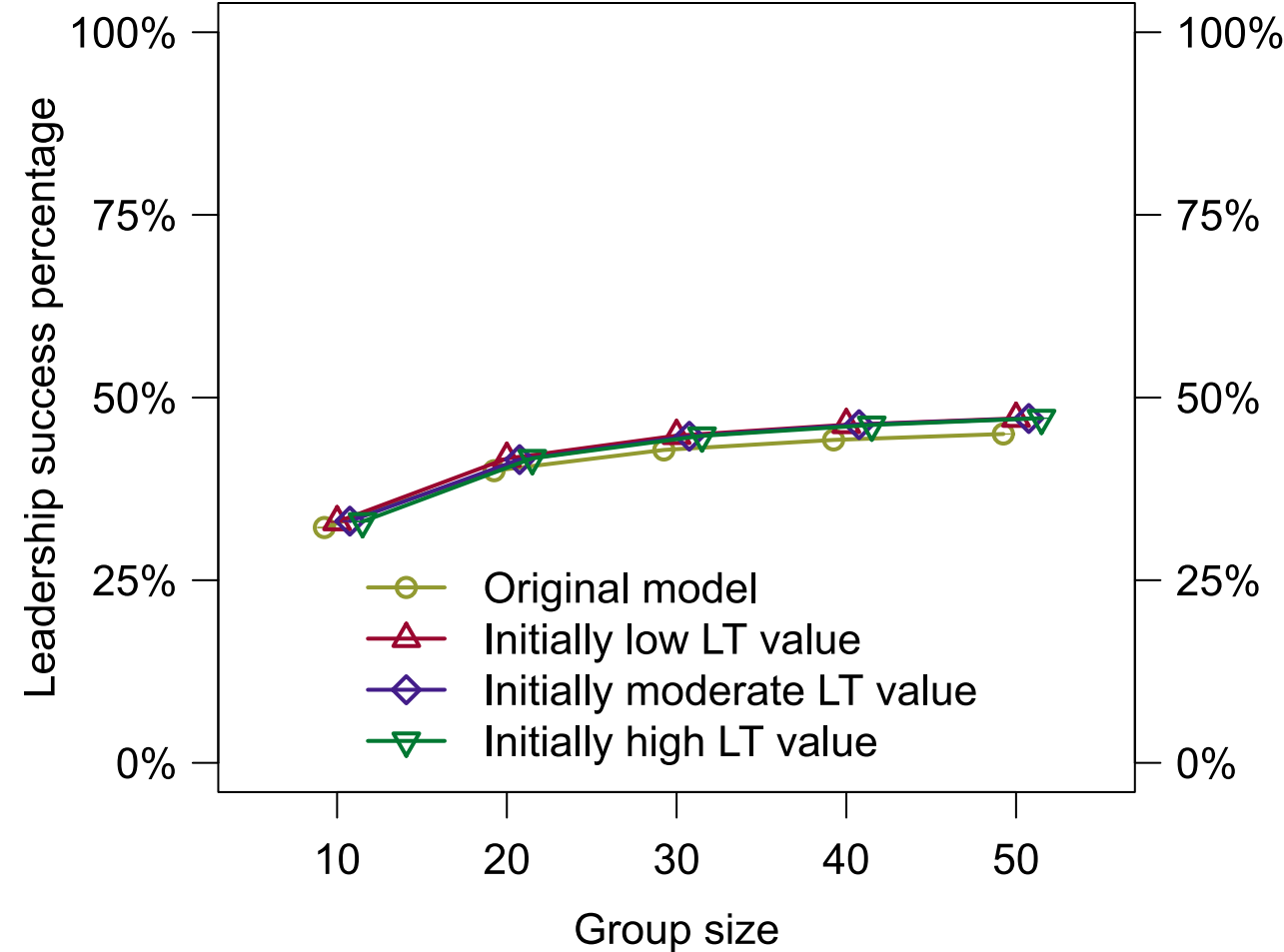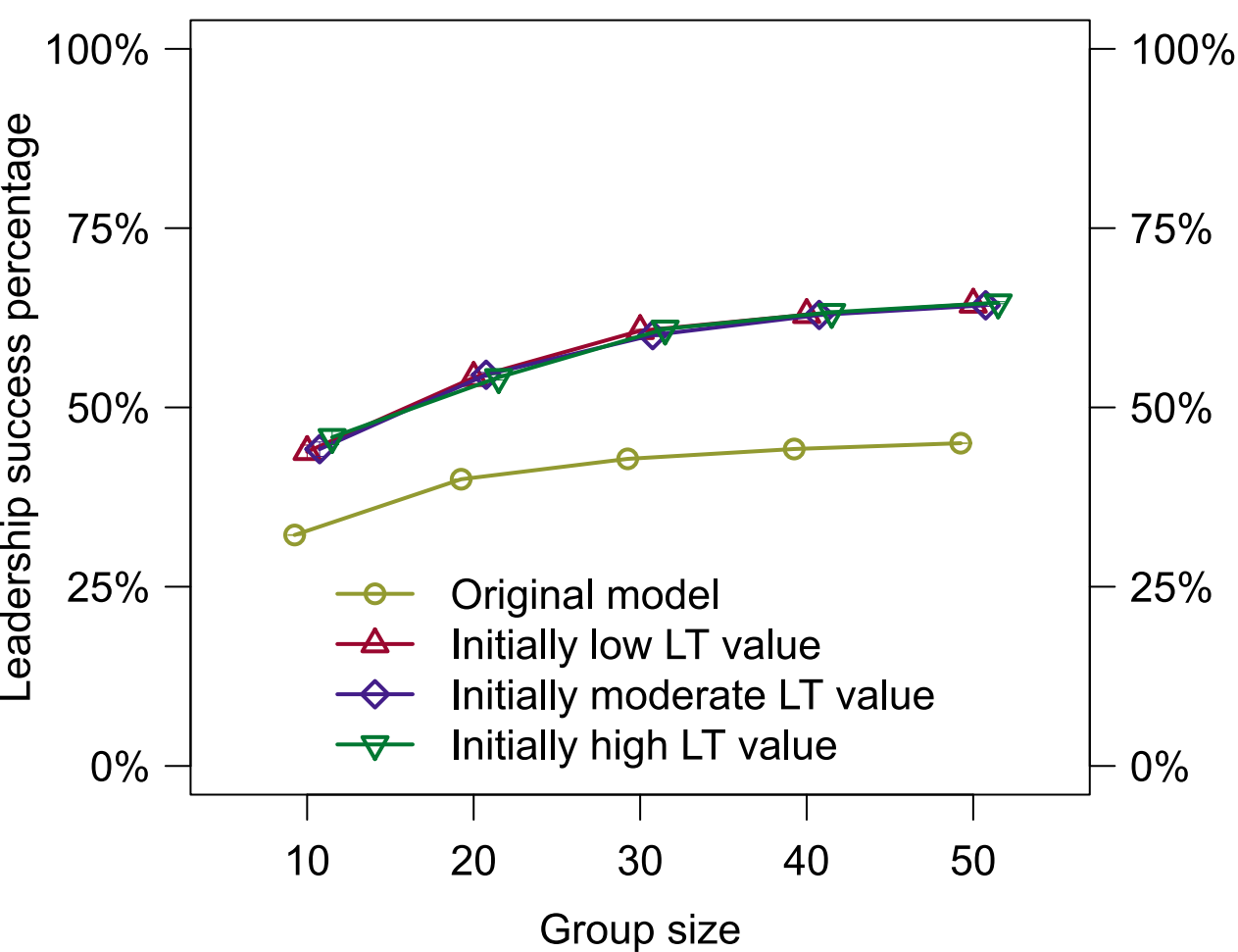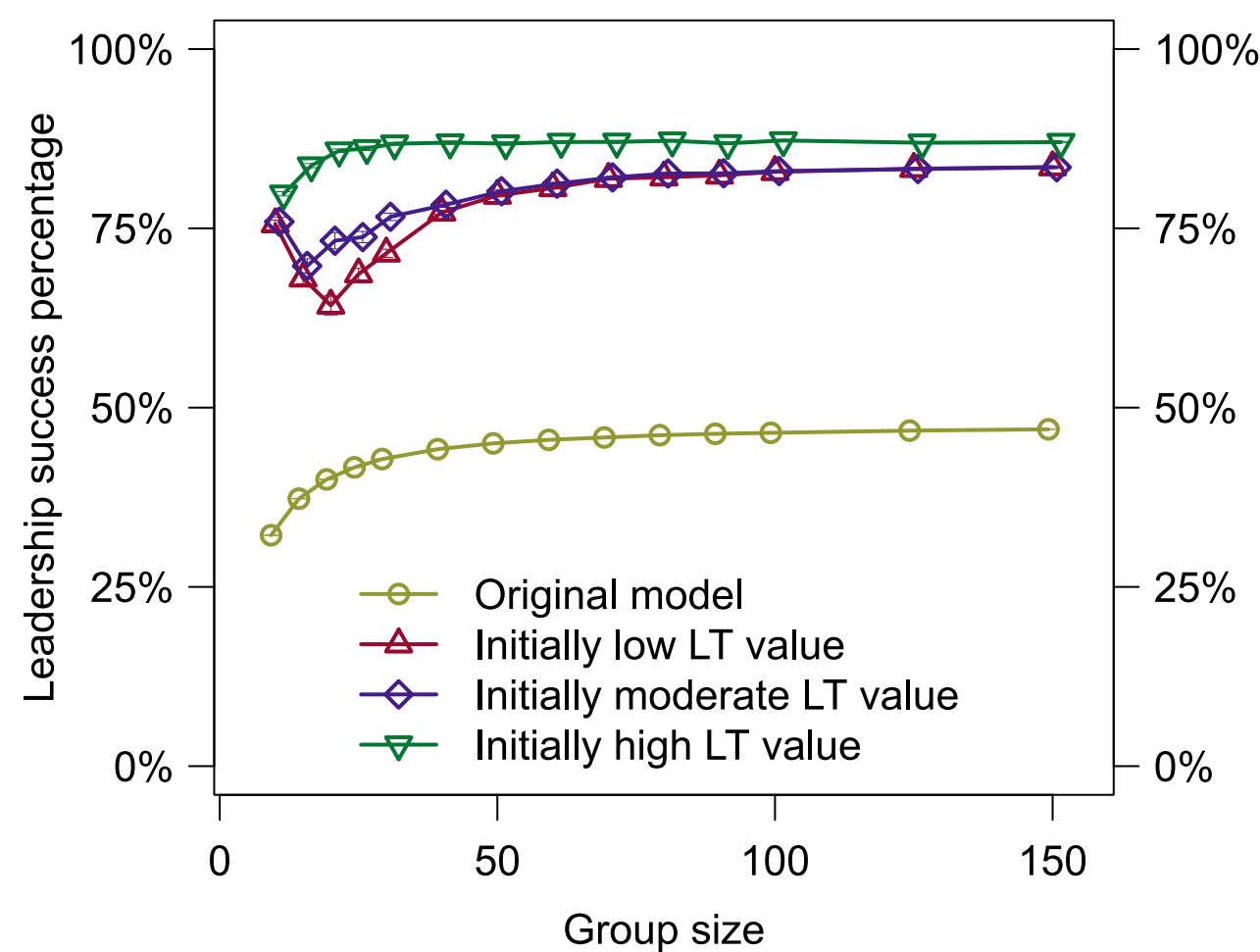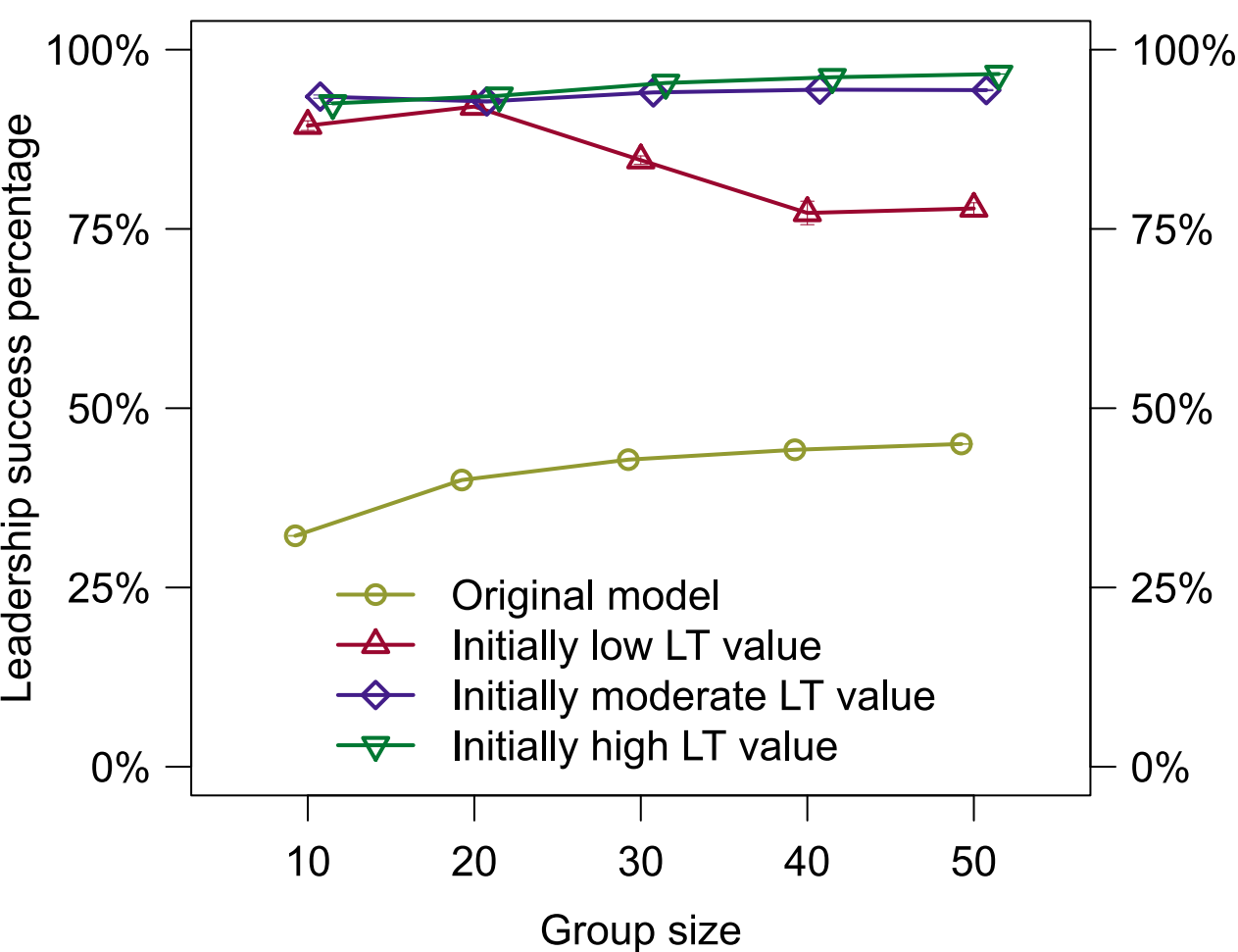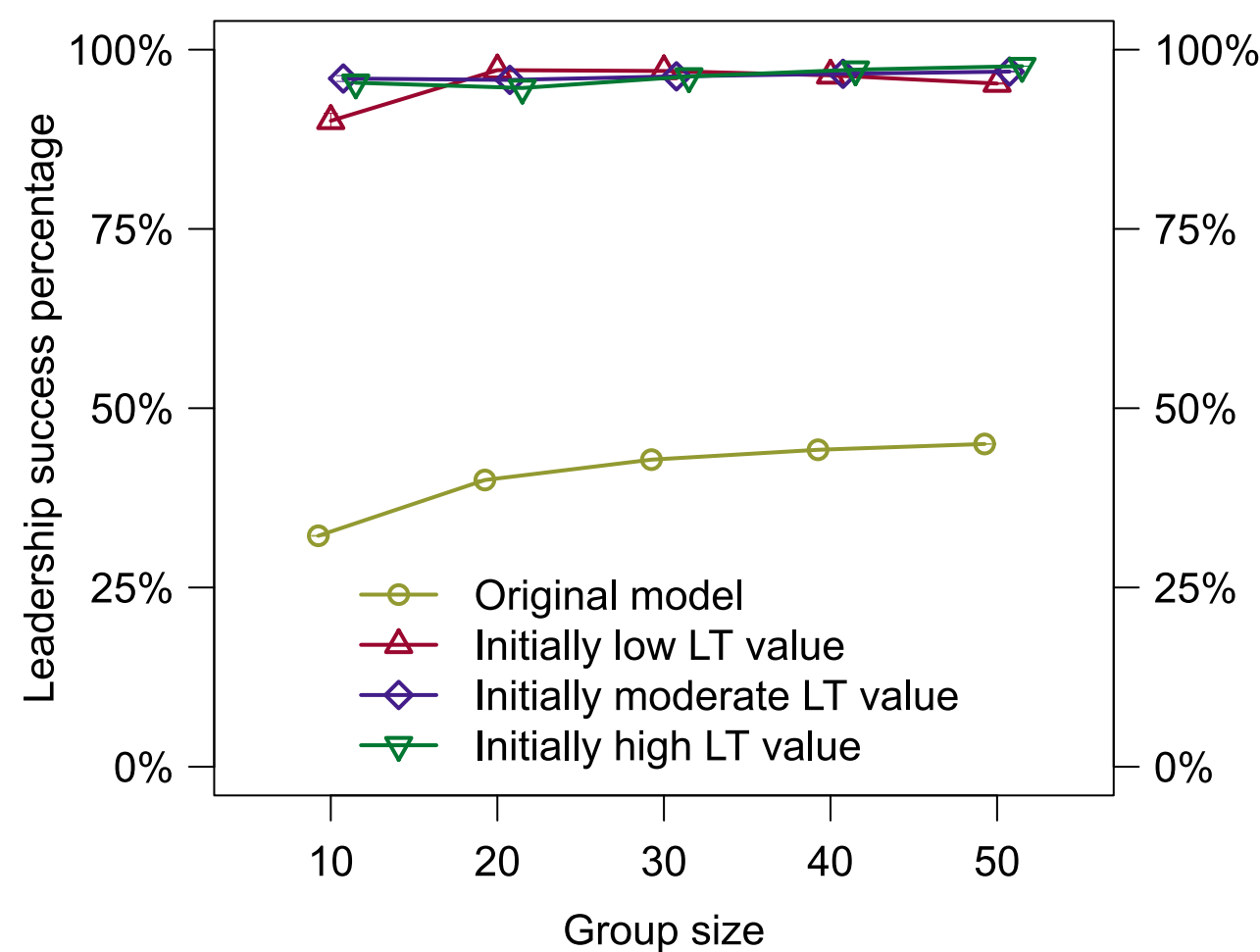

Supplement: S4 Fig — (a) A linear function. (b)-(f) A sigmoid function with different a coefficients. (d) Sigmoid function used in the principal paper. (PDF) [file pone.0134222.s004.pdf]
