# Supplementary material for: Emergence of Leadership within a Homogeneous Group
Source: PLoS One. 2015 Jul 30;10(7):e0134222. doi: 10.1371/journal.pone.0134222 (PMC4520564; doi:10.1371/journal.pone.0134222)
Supplement: S1 Table — The algorithm was developed in conjunction with the decision-making model for the original work and was rewritten for use in this work. (PDF) [file pone.0134222.s005.pdf]

Table S1. The collective movement simulation algorithm. The algorithm was developed in conjunction with the movement model for the original work and was rewritten for use in this work.

```
Leader ← EarliestInitiator( Group )                              ▷ Get the initiator
Departed ← {Leader}
Remaining ← Group − {Leader}
while Remaining ≠ ∅ do                                          ▷ Process all the nonparticipants
    Follower ← FindEarliestFollower( Remaining )                ▷ Get the first follower
    cancelTime ← CalcCancelTime( Leader )
    if Follower follows before Leader cancels then              ▷ Which happens first?
        Departed ← Departed + {Follower}
        Remaining ← Remaining − {Follower}
    else
        break                                                   ▷ Leader cancels before follower follows
    end if
end while
```
