# Supplementary material for: Emergence of Leadership within a Homogeneous Group
Source: PLoS One. 2015 Jul 30;10(7):e0134222. doi: 10.1371/journal.pone.0134222 (PMC4520564; doi:10.1371/journal.pone.0134222)
Supplement: S2 Table — (PDF) [file pone.0134222.s006.pdf]

Table S2. A full statistical analysis of the mean success percentage of initiators.

Group Size	None	Low	Moderate	High	Low vs. Moderate	Low vs. High	Moderate vs. High
10	0.322 ± 0.000	0.756 ± 0.006	0.759 ± 0.006	0.798 ± 0.003	0.693	< 0.001	< 0.001
15	0.373 ± 0.000	0.680 ± 0.007	0.698 ± 0.010	0.838 ± 0.003	0.169	< 0.001	< 0.001
20	0.400 ± 0.000	0.643 ± 0.013	0.733 ± 0.011	0.858 ± 0.002	< 0.001	< 0.001	< 0.001
25	0.417 ± 0.000	0.686 ± 0.008	0.738 ± 0.008	0.862 ± 0.003	< 0.001	< 0.001	< 0.001
30	0.428 ± 0.000	0.715 ± 0.006	0.766 ± 0.005	0.868 ± 0.002	< 0.001	< 0.001	< 0.001
40	0.442 ± 0.000	0.772 ± 0.003	0.783 ± 0.004	0.869 ± 0.002	0.018	< 0.001	< 0.001
50	0.450 ± 0.000	0.796 ± 0.002	0.802 ± 0.002	0.868 ± 0.002	0.031	< 0.001	< 0.001
60	0.455 ± 0.000	0.806 ± 0.001	0.812 ± 0.002	0.870 ± 0.002	0.013	< 0.001	< 0.001
70	0.458 ± 0.000	0.819 ± 0.001	0.821 ± 0.001	0.871 ± 0.002	0.334	< 0.001	< 0.001
80	0.462 ± 0.000	0.821 ± 0.001	0.826 ± 0.001	0.872 ± 0.002	0.004	< 0.001	< 0.001
90	0.463 ± 0.000	0.824 ± 0.001	0.827 ± 0.001	0.869 ± 0.001	0.041	< 0.001	< 0.001
100	0.465 ± 0.000	0.829 ± 0.001	0.830 ± 0.001	0.873 ± 0.001	0.491	< 0.001	< 0.001
125	0.468 ± 0.000	0.833 ± 0.001	0.833 ± 0.001	0.869 ± 0.001	0.732	< 0.001	< 0.001
150	0.470 ± 0.000	0.835 ± 0.001	0.835 ± 0.001	0.870 ± 0.001	0.818	< 0.001	< 0.001
