# Supplementary material for: Emergence of Leadership within a Homogeneous Group
Source: PLoS One. 2015 Jul 30;10(7):e0134222. doi: 10.1371/journal.pone.0134222 (PMC4520564; doi:10.1371/journal.pone.0134222)
Supplement: S3 Table — (PDF) [file pone.0134222.s007.pdf]

Table S3. A full statistical analysis of the mean percentage of initiation attempts required for differentiation.

**Group Size**	**Low**	**Moderate**	**High**	**Low vs. Moderate**	**Low vs. High**	**Moderate vs. High**
10	0.13 ± 0.01	0.13 ± 0.01	0.17 ± 0.03	0.60	0.37	0.26
15	0.06 ± 0.00	0.07 ± 0.01	0.11 ± 0.01	0.28	< 0.01	< 0.01
20	0.08 ± 0.02	0.06 ± 0.00	0.10 ± 0.01	0.14	0.31	< 0.01
25	0.09 ± 0.01	0.07 ± 0.01	0.08 ± 0.00	0.36	0.45	0.62
30	0.10 ± 0.01	0.07 ± 0.01	0.08 ± 0.00	0.02	0.05	0.19
40	0.14 ± 0.01	0.11 ± 0.01	0.08 ± 0.00	0.02	< 0.01	< 0.01
50	0.14 ± 0.01	0.11 ± 0.01	0.08 ± 0.00	< 0.01	< 0.01	< 0.01
60	0.14 ± 0.01	0.13 ± 0.01	0.09 ± 0.00	0.03	< 0.01	< 0.01
70	0.14 ± 0.00	0.13 ± 0.00	0.09 ± 0.00	0.02	< 0.01	< 0.01
80	0.15 ± 0.00	0.13 ± 0.00	0.09 ± 0.00	< 0.01	< 0.01	< 0.01
90	0.15 ± 0.00	0.14 ± 0.00	0.09 ± 0.00	0.01	< 0.01	< 0.01
100	0.15 ± 0.00	0.13 ± 0.00	0.09 ± 0.00	< 0.01	< 0.01	< 0.01
125	0.15 ± 0.00	0.14 ± 0.00	0.09 ± 0.00	< 0.01	< 0.01	< 0.01
150	0.15 ± 0.00	0.14 ± 0.00	0.09 ± 0.00	< 0.01	< 0.01	< 0.01
