# Supplementary material for: Emergence of Leadership within a Homogeneous Group
Source: PLoS One. 2015 Jul 30;10(7):e0134222. doi: 10.1371/journal.pone.0134222 (PMC4520564; doi:10.1371/journal.pone.0134222)
Supplement: S4 Table — (PDF) [file pone.0134222.s008.pdf]

Table S4. A full statistical analysis of low to high LT value transitions.

Group Size	Low	Moderate	High	Low vs. Moderate	Low vs. High	Moderate vs. High
10	0.070 ± 0.007	0.048 ± 0.007	0.004 ± 0.003	0.025	< 0.001	< 0.001
15	0.015 ± 0.004	0.000 ± 0.000	0.000 ± 0.000	0.002	0.002	1
20	0.013 ± 0.003	0.001 ± 0.001	0.000 ± 0.000	< 0.001	< 0.001	0.322
25	0.019 ± 0.003	0.005 ± 0.002	0.000 ± 0.000	< 0.001	< 0.001	0.032
30	0.023 ± 0.003	0.007 ± 0.002	0.000 ± 0.000	< 0.001	< 0.001	0.001
40	0.054 ± 0.004	0.025 ± 0.004	0.000 ± 0.000	< 0.001	< 0.001	< 0.001
50	0.068 ± 0.004	0.021 ± 0.003	0.000 ± 0.000	< 0.001	< 0.001	< 0.001
60	0.064 ± 0.004	0.034 ± 0.003	0.001 ± 0.000	< 0.001	< 0.001	< 0.001
70	0.069 ± 0.003	0.042 ± 0.003	0.001 ± 0.001	< 0.001	< 0.001	< 0.001
80	0.074 ± 0.003	0.039 ± 0.002	0.002 ± 0.001	< 0.001	< 0.001	< 0.001
90	0.077 ± 0.004	0.046 ± 0.003	0.004 ± 0.001	< 0.001	< 0.001	< 0.001
100	0.077 ± 0.003	0.043 ± 0.003	0.002 ± 0.001	< 0.001	< 0.001	< 0.001
125	0.078 ± 0.002	0.047 ± 0.002	0.006 ± 0.001	< 0.001	< 0.001	< 0.001
150	0.076 ± 0.002	0.048 ± 0.002	0.005 ± 0.001	< 0.001	< 0.001	< 0.001
