# Supplementary material for: Emergence of Leadership within a Homogeneous Group
Source: PLoS One. 2015 Jul 30;10(7):e0134222. doi: 10.1371/journal.pone.0134222 (PMC4520564; doi:10.1371/journal.pone.0134222)
Supplement: S1 Text — Additional simulations were performed using fixed LT values to ensure that the modification made to add LT values to the collective decision-making model did not bias the model towards one particular LT value. (PDF) [file pone.0134222.s009.pdf]

S1 Text

Emergence of Leadership within a Homogeneous Group

Eskridge, Valle, Schlupp

To ensure that the modification made to add leadership tendency (LT) values the collective decision-making model did not bias the model towards one particular LT value, simulations were performed in which all individuals within the group were given the same, fixed LT value. LT values of 0.10, 0.20, 0.35, 0.50, 0.65, 0.80, and 0.90 and group sizes of 10, 20, 30, 40, and 50 were simulated. Fig. S1 shows that there was no difference in the leadership success in collective movement initiations between different LT values for a given group size. In fact, when the same random seed was used, the results of simulations with different LT values and the same group size were identical, despite the differences in LT value. This indicates that the modifications made to the decision rate equations in the model to incorporate LT values were balanced and did not favor one value over another. For example, although individuals with high LT values are less likely to cancel a movement, they are also less likely to follow an initiator. Similarly, although individuals with low LT values are more likely to follow an initiator, they are also more likely to cancel a movement.
