# Supplementary material for: Emergence of Leadership within a Homogeneous Group
Source: PLoS One. 2015 Jul 30;10(7):e0134222. doi: 10.1371/journal.pone.0134222 (PMC4520564; doi:10.1371/journal.pone.0134222)
Supplement: S2 Text — This text presents supporting results regarding the challenge in developing a model in which LT value differentiation is stable in a constant environment. (PDF) [file pone.0134222.s010.pdf]

S2 Text

Emergence of Leadership within a Homogeneous Group

Eskridge, Valle, Schlupp

This document presents supporting results regarding the challenge in developing a model in which leadership tendency (LT) value differentiation is stable in a constant environment. A discussion of the configuration of numerical simulations using the collective decision-making model in which stable LT values did not emerge is provided. Also provided are some of the results of these simulations, including representative histories of LT value changes throughout an evaluation.

Numerical Implementation

The differentiation results from the principal paper were notable in that differentiation was able to occur and the emerged LT values were stable. Exploratory simulations showed that these properties were not typical for all systems using the model discussed. They were highly dependent on the type of function used to calculate the "k factor." The general form of the sigmoid function in Equation 5 from the principal paper is the following:

$$k = 2\left(1 + \mathrm{e}^{(0.5-p')\times a}\right)^{-1} \tag{1}$$

where a determines the slope at the inflection point and p' is p for initiating decisions and $1-p$ for canceling and following decisions. In the principal paper, $a = 10$ was used for all the simulations presented and resulted differentiation and stable LT values. To illustrate the effect of this value on the overall system, alternative values of $a = 5, 7, 15, 20$ were evaluated (see Fig. S2).For comparison purposes, a linear function was also used:

$$k = 2p' \tag{2}$$

where p' is, again, p for initiating decisions and $1-p$ for canceling and following decisions. As with the simulations in the principal paper, treatments were performed using all individual LT values initialized to the same following values: low ($L_i = 0.2$), moderate ($L_i = 0.5$), and high ($L_i = 0.8$). Since most of the variations in performance and LT value distribution observed in the principal paper occurred for smaller group sizes, group sizes ranging only from 10 to 50 agents were used, as opposed to the range of 10 to 150 in the principal paper. Fifty evaluations were performed for each group size, each with a different random seed. A single evaluation consisted of $2,000 \times N$ movement attempts, where N was the group size.

Results

Fig. S3 shows representative histories of LT value changes using the alternative methods of calculating the "k factor" described above. In simulations using the linear function (see Fig. S3(a)) or the sigmoid function with $a = 5$ (see Fig. S3(b)), individual LT values did not differentiate into high and low. In simulations using the sigmoid function with $a = 7$ (see Fig. S3(c)), individual LT values were able to differentiate, but the LT values were not very stable and fluctuated throughout the evaluation. Lastly, in simulations using larger the sigmoid function with larger a values (see Fig. S3(d)–S3(f)), individual LT values differentiated and were stable.

Fig. S4 shows the mean leadership success for the alternative methods of calculating the "k factor" described above. Without the differentiation of LT values, simulations using the linear function (see Fig. S4(a)) or the sigmoid function with $a = 5$ (see Fig. S4(b)) were not able to perform better than simulations that did not use LT values. Only once individual LT values began to differentiate (see Fig. S4(c)–S4(f)) did leadership success improve. Simulations using $a = 15$ and starting with an initially low LT value (see Fig. S4(f)) exhibited a drop in leadership success as group size increased. At present, we do not know what caused this drop and further investigation is warranted.
